# Prognostic value of new left atrial volume index severity partition cutoffs after cardiac rehabilitation program in patients undergoing cardiac surgery

**DOI:** 10.1186/s12947-016-0077-0

**Published:** 2016-08-23

**Authors:** Davide Lazzeroni, Nicola Gaibazzi, Matteo Bini, Giacomo Bussolati, Umberto Camaiora, Roberto Cassi, Simone Geroldi, Pietro Tito Ugolotti, Lorenzo Brambilla, Valerio Brambilla, Paolo Castiglioni, Paolo Coruzzi

**Affiliations:** 1 Fondazione Don Carlo Gnocchi, University of Parma, Fondazione Don Gnocchi, Piazzale dei servi n° 3, 43121 Parma, Italy; 2 Department of Cardiology, Parma University Hospital, Parma, Italy; 3 Department of Clinical and Experimental Medicine, University of Parma, Parma, Italy; 4 IRCCS Fondazione Don C. Gnocchi, Milan, Italy

**Keywords:** Left atrial volume index, Coronary artery by-pass graft, Cardiac valve surgery, Echocardiography, cardiovascular outcomes

## Abstract

**Background:**

Previous studies showed that left atrial enlargement is an independent marker of adverse outcomes in both primary and secondary cardiovascular prevention. However, no data are available on long-term outcomes in patients undergoing valve surgery and/or coronary artery by-pass graft (CABG) surgery. Aim of the study was to evaluate long-term prognostic role of left atrial volume index (LAVi) after cardiac surgery, using the cutoff values recently proposed by the European Association of Cardiovascular Imaging and American Society of Echocardiography.

**Methods:**

We created a retrospective registry of 1703 consecutive patients who underwent cardiovascular rehabilitation program after cardiac surgery, including CABG, valve surgery and valve + CABG surgery. LAVi was calculated as ratio of left atrium volume to body surface area, in ml/m^2^ at discharge; 563 patients with available LAVi data were included in the study.

**Results:**

In the whole population LAVi was 36 ± 14 ml/m^2^ (mean ± SD) and the follow-up time was 5 ± 1.5 years. Increased LAVi (>34 ml/m^2^) predicted major adverse cardiovascular and cerebrovascular events (MACCEs) (HR = 2.1; CI95 %: 1.4–3.1; *p* < 0.001) and cardiovascular mortality (HR = 2.2; CI95 %: 1.0–4.5; *p* = 0.032). An increased LAVi remained MACCEs predictor after adjustement for age, gender, diabetes, atrial fibrillation at discharge, echocardiographic E/A ratio and left ventricular ejection fraction (HR = 1.8; CI95 %: 1.0–3.0; *p* = 0.036). When the study population was split according to increasing LAVi values, left atrium enlargement resulted a predictor of progressively worse adverse outcome.

**Conclusions:**

LAVi is a predictor of long-term adverse cardiovascular outcome after cardiac surgery, even after correction for main clinical and echocardiographic variables. The recently recommended LAVi severity cutoffs appear adequate to effectively stratify outcome in patients undergoing rehabilitation after cardiac surgery.

## Background

Left atrium (LA) modulates left ventricular (LV) filling and cardiovascular performance by functioning as a reservoir and a conduit for pulmonary venous return and as a “booster” that augments ventricular filling during late ventricular diastole. LA also reflects LV filling pressure and is capable of remodeling (enlarging) in response to its elevation [1, 2]. Although there is a growing body of evidence demonstrating that LA enlargement is an independent marker of adverse outcomes both in primary [3–7] and secondary [8–11] cardiovascular prevention, to date, there are no published data assessing long-term outcome in patients undergoing valve and coronary artery by-pass graft (CABG) surgery.

Aim of this retrospective study was to examine long-term prognostic implications of left atrial volume index (LAVi) assessment in patients undergoing cardiovascular rehabilitation program (CRP) after cardiac surgery, using the cutoff values proposed by the recently-published guidelines of the European Association of Cardiovascular Imaging (EACVI) and American Society of Echocardiography (ASE) [12].

## Methods

### Patients’ selection

We created a registry of consecutive patients undergoing cardiovascular rehabilitation program (CRP) after cardiac surgery, including CABG, valve surgery and combined valve and CABG surgery, from January 2007 to June 2012. A total of 1703 subjects (70 % men, mean age of 69 ± 11 years) were collected and, out of them, all 563 patients with available biplane left atrial volume data were extracted for the current analysis. The internal review board of Fondazione Don Gnocchi approved data collection. All patients completed a standard in-hospital CRP, lasting approximately 2 weeks, consisting of supervised exercise sessions (120 min per day), lifestyle and risk factor management, medical counseling and medical therapy optimization. For each patient we collected variables from medical record including anamnestic and demographic findings, clinical and laboratory markers, electrocardiographic and echocardiographic measurements, physical activity data, pharmacological therapy adherence. Outcomes were collected through a telephonic questionnaire administered by a medical doctor or a supervised fellow and all patients provided oral informed consent. End points were: overall mortality and major adverse cardiovascular and cerebrovascular events (MACCEs) defined as either: cardiovascular (CV) mortality, non-fatal acute coronary syndrome (ACS), heart failure (HF) and stroke.

### Echocardiographic data

M-mode and 2-dimensional echocardiographic images were obtained with a commercially available echocardiography machine (Esaote MyLab™ 60). LA volume was calculated using the biplane area-length method at discharge of CRP [12]. Measurements were obtained in end-systole from the frame preceding mitral valve opening. LAVi was calculated as the ratio of LA volume to body surface area (ml/m^2^). In agreement with ASE and EACVI guidelines, we assumed LAVi > 34 ml/m^2^ as the LA enlargement threshold and we further classified LAVi values from 35 to 41 ml/ m^2^ as mild LA enlargement, from 42 to 48 ml/ m^2^ as moderate LA enlargement and greater than 48 ml/ m^2^ as severe enlargement [12].

### Statistical analysis

Continuous variables were expressed as mean (M) and standard deviation (SD), categorical variables as number (N) and percentage (%). The Student 2-sample *t* test, Pearson *χ*2 test, Mann–Whitney–Wilcoxon rank-sum test were used to compare the differences between groups for continuous, categorical and ordinal variables, respectively. Cox proportional hazard regression analysis was performed to create survival-free-from-event curves. Multivariate Cox regression analysis was used to create adjustment of hazard ratio (HR) for age, left ventricular ejection fraction (LVEF), gender, diabetes, and ratio of the early (E) to late (A) ventricular filling velocities (E/A ratio). Event-free survival analysis was measured from admission to the first event. Significance was defined as a *p* value <0.05. All statistics were performed with SPSS version 21 (IBM Corporation).

## Results

### Demographic and clinical characteristics

A total of 563 patients were included in the study. Mean age was 69 ± 10 years and male gender was prevalent, with 399 males (70 %). 149 patients (26 %) were affected by type 2 diabetes and 305 patients (54 %) by arterial hypertension. 292 patients (52 %) underwent CABG, 191 (34 %) valve surgery, 80 (14 %) combined valve and CABG surgery.

One hundred and eighty-four patients (33 %) underwent cardiac revascularization after ACS and 98 (18 %) after stable angina or silent ischemia; 100 patients (18 %) were affected by mitral valve regurgitation, 9 (2 %) by mitral valve stenosis, 73 (13 %) by aortic valve stenosis and 41 (7 %) by aortic valve regurgitation. LAVi and LVEF mean values over the whole group of patients were 36 ± 14 ml/m^2^ and 51 ± 9 %, respectively.

LA enlargement was found in 257 patients (46 %), 97 (17 %) patients with mild LA enlargement, 71 (13 %) with moderate LA enlargement and 89 (16 %) with severe LA enlargement.

Patients with LA enlargement were significantly older compared with patients with normal LA (71 ± 9 vs 68 ± 10 years, <*p* = 0.001). A higher prevalence of normal LA was observed in the subgroup of patients with established coronary artery disease such as prior acute coronary syndrome (normal LA 63 % vs LA enlargement 37 %; *p* = 0.001) or stable angina (normal LA 67 % vs LA enlargement 33 %; *p* = 0.002). Conversely, LA enlargement was more frequent in the subgroup of patients with mitral regurgitation (normal LA 44 % vs LA enlargement 56 %; *p* = 0.034) and aortic stenosis (normal LA 32 % vs LA enlargement 68 %; *p* < 0.001). Mean LVEF was significantly lower in patients with LAVi > 34 ml/m^2^ in comparison to the group with normal LAVi (49 ± 10 % vs 52 ± 9 %; *p* < 0.005). Sinus rhythm rate at discharge was higher in patients with normal LAVi (59 % vs 41 %; *p* < 0.01) and no differences in the use of beta-blockers (52 % vs 48 %; *p* = n.s.) and amiodarone (51 % vs 49 %; *p* = n.s.) were found between normal LAVi and LAVi enlargement; conversely, statins and ace-inhibitors administration was higher in patients with normal LAVi (56 % vs 44 %; *p* < 0.01 and 57 % vs 43 %; *p* < 0.01 respectively).

Table 1 shows differences in baseline parameters between normal LA and LA enlargement.

**Table 1:** Baseline characteristics

Parameters	Normal LA (LAVi ≤ 34 ml/m2)	LA Enlargement (LAVi > 34 ml/m2)
	*N* = 306	*N* = 257
Age (years)	68 (10)	71 (9)**
Male sex	245 (61)	154 (39)**
BMI (kg/m^2^)	26.9 (4)	26.7 (4)
BSA (m^2^)	1.9 (0.2)	1.8 (0.2)**
CV risk Factors		
Familiar history (CVD)	159 (56)	120 (44)
AHT	159 (52)	146 (48)
DM	83 (55)	66 (45)
Dyslipidaemia	165 (57)	120 (43)
Types of cardiac surgery		
CABG	197 (75)	95 (25)**
Heart-valve surgery	73 (25)	118 (75)**
CABG + Valve surgery	37 (46)	43 (54)
Clinical indications		
ACS	116 (63)	68 (37)*
Stable Angina	66 (67)	32 (33)*
MV Stenosis	2 (22)	7 (78)
MV regurgitation	44 (44)	56 (56)*
AV Stenosis	23 (32)	20 (68)**
AV regurgitation	19 (46)	22 (54)
Other indication	36 (62)	22 (38)
Echocardiographic parameters		
LAV (ml)	49 (11)	85 (22)**
LAd (mm)	38 (5)	54 (6)**
LVEF (%)	52 (9)	49 (10)*
LV EDV (ml)	105 (41)	108 (42)
IVS (mm)	12 (2)	13 (2)**
E/A Ratio	1 (0.4)	1 (0.6)
Medications		
β-Blocker	201 (52)	185 (48)
Amiodarone	79 (51)	77 (49)
OAT	72 (38)	117 (62)**
ACEi	216 (57)	170 (43)**
Statins	218 (56)	168 (44)**

Values are expressed as mean and standard deviation or as number and percentage

*LA* left atrium; *LAVi* left atrial volume index; *BMI* body mass index; *CVD* cardiovascular disease; *AHT* arterial hypertension; *DM* diabetes mellitus; *CABG* coronary artery bypass graft; *ACS* acute coronary syndrome; *MV* mitral valve; *AV* aortic valve; *Lad* left atrium antero-posteior diameter; *LVEF* left ventricular ejection fraction; *IVSs* interventricular septum; *LV EDV* left ventricular end-diastolic volume; *OAT* oral anticoagulant therapy

*and **indicate differences at significance levels p <0.05 and p <0.01 respectively

### End point

Mean follow-up was 5 ± 1.5 years. All-cause mortality consisted in 55 deaths (10 %) and the total amount of MACCEs was 114 (20 %), composed by 31 CV deaths (5 %), 43 HF hospitalization (8 %), 15 strokes (3 %) and 25 ACS (4 %).

**Fig. 1:**
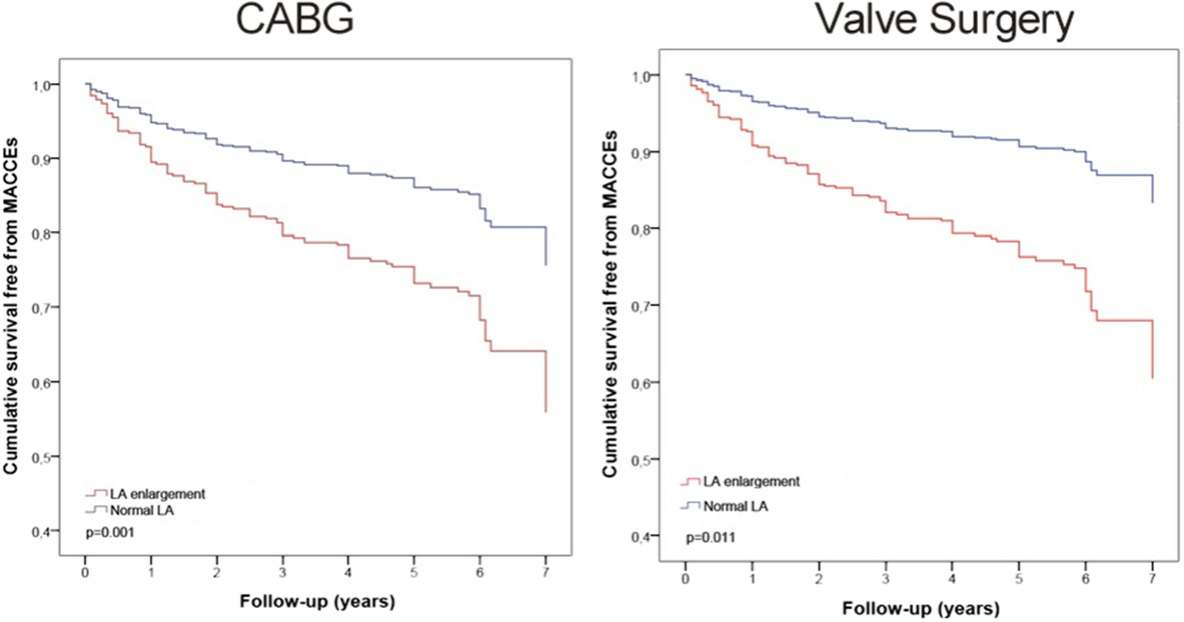
*Left*: LA enlargement and survival free from MACCEs in CABG patients. *Right*: LA enlargement and survival free from MACCEs in valve surgery patients. *MACCEs, major adverse cardiovascular and cerebrovascular events; LA, left atrium*

MACCEs were 2.1-fold higher in patients with LAVi > 34 ml/m^2^ compared with patients with normal LA volume, this difference being significant even considering patients who received CABG or valve surgery separately (in particular in patients with mitral and aortic regurgitation; see Table 2). Moreover, event-free survival from MACCEs was significantly lower in both patients who underwent CABG and valve surgery with enlarged LA (Fig. 1).

**Table 2:** HR for primary and secondary end points by univariate Cox proportional hazard regression analysis

			LA severity partition cut-off					
	LA enlargement (LAVi > 34 ml/m2)		Mild LA enlargement		Moderate LA enlargement		Severe LA enlargement	
MACCEs	HR (95 % CI)	p	HR (95 % CI)	p	HR (95 % CI)	p	HR (95 % CI)	p
All Patients	2.1 (1.4–3.1)	0.000#	1.2 (0.7–2.2)	0.457	2.2 (1.3–2.7)	0.003*	3.1 (2.0–4.9)	0.000#
CABG Patients	2.1 (1.3–3.2)	0.001*	1.8 (0.7–4.5)	0.201	3.2 (1.2–7.9)	0.013*	6.1 (2.6–14.1)	0.000#
Valve Patients	2.7 (1.2–6.0)	0.011*	1.6 (0.5–4.9)	0.402	2.9 (1.1–7.7)	0.025*	3.3 (1.4–8.0)	0.005#
Aortic stenosis	6.6 (0.8–51)	0.071	0.1 (0.1–1.0)	0.521	1.9 (0.3–11)	0.482	1.6 (0.2–9.6)	0.060
Aortic regurgitation	4.2 (1.5–11)	0.004*	3.3 (0.7–15)	0.117	3.7 (1.1–12)	0.028*	4.5 (1.5–13)	0.006*
Mitral regurgitaton	4.2 (1.5–11)	0.004*	1.9 (0.1–31)	0.638	6.4 (0.7–57)	0.096	7.0 (0.9–57)	0.067
Valve + CABG	2.3 (0.9–5.6)	0.053	1.6 (0.4–6.3)	0.484	2.4 (0.7–7.8)	0.122	2.6 (0.9–7.1)	0.050
All-cause mortality	1.6 (0.9–2.8)	0.064	1.2 (0.5–2.7)	0.533	1.6 (0.7–3.5)	0.258	2.2 (1.1–4.2)	0.025*
CV mortality	2.2 (1.0–4.5)	0.032*	1.2 (0.4–3.7)	0.739	2.5 (0.9–6.6)	0.070	3.1 (1.3–7.4)	0.010*
Heart failure	1.8 (0.9–3.5)	0.057	0.8 (0.2–2.5)	0.796	2.5 (1.0–5.8)	0.034*	2.5 (1.1–5.4)	0.023*
Stroke	1.6 (0.5–4.8)	0.327	1.0 (0.2–5.3)	0.918	n.a.	n.a.	3.8 (1.2–11.9)	0.019*
					n.a.			
ACS	1.9 (0.8–4.4)	0.137	1.5 (0.4–4.9)	0.492	1.6 (0.4–5.9)	0.483	2.6 (0.9–7.4)	0.067

*MACCEs* major adverse cardiovascular and cerebrovascular events; *LA* left atrial; *LAVi* left atrial volume index; *HR* hazard ratio; *CI* confidence interval; *CABG* coronary artery by-pass graft; *Valve* valve surgery; *CV* cardiovascular; *ACS* acute coronary syndrome; *n.a* not applicable (no event)

*P* value: difference in comparison with normal LAVi group (≤34 ml/m2) * *p* < 0.05; # *p* < 0.001

**Fig. 2:**
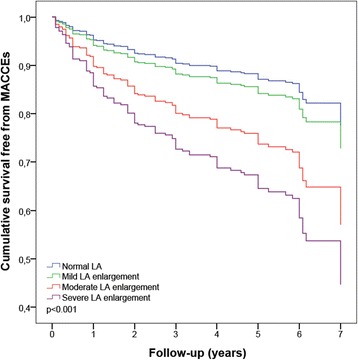
MACCEs and LA severity partition cut-offs after cardiac surgery. *MACCEs, major adverse cardiovascular and cerebrovascular events; LA, left atrium*

**Fig. 3:**
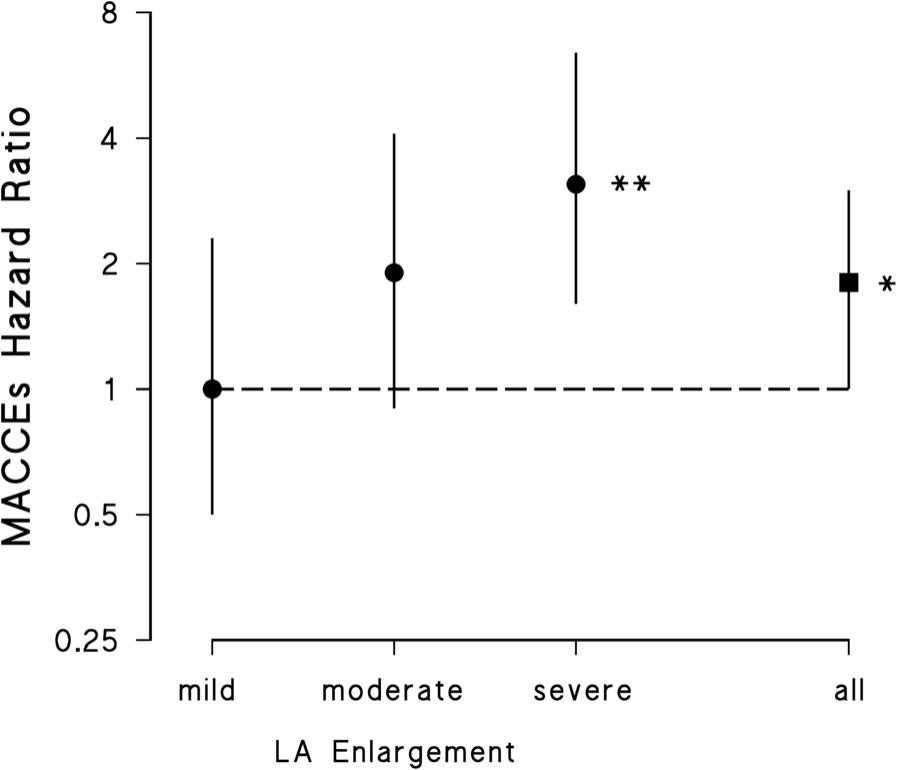
Hazard Ratio with 95 % CI for MACCEs calculated after adjustment for age, LVEF, gender, diabetes, atrial fibrillation at discharge and E/A ratio, in subgroups of patients with mild, moderate and severe LA enlargement, and in all patients with LA enlargement; * and ** indicate significant differences from HR = 1 at *p* < 0.05 and *p* < 0.01 significance levels

By dividing the whole population according to severity partition cut-offs, MACCEs rate was significantly higher in patients with either moderate or severe LA enlargement but not in those with mild LA enlargement (Fig. 2 and Table 2). Multivariate analysis, adjusted for age, gender, diabetes, E/A, atrial fibrillation at discharge and LVEF, confirmed the role of LA enlargement in predicting MACCEs in the whole population; moreover, the progressive increase of LAVi severity maintained an incremental risk of MACCEs in patients with LA enlargement ranging from moderate to severe (Fig. 3).

Severe LA enlargement resulted a significant and robust predictor of any type of secondary end point: all-cause mortality, CV mortality, HF and stroke. In patients with moderate LA enlargement, HF rate was 2.5-fold higher in comparison with normal LA size group. Outcomes of patients with normal LAVi and with mild LA enlargement did not differ (Table 2).

**Fig. 4:**
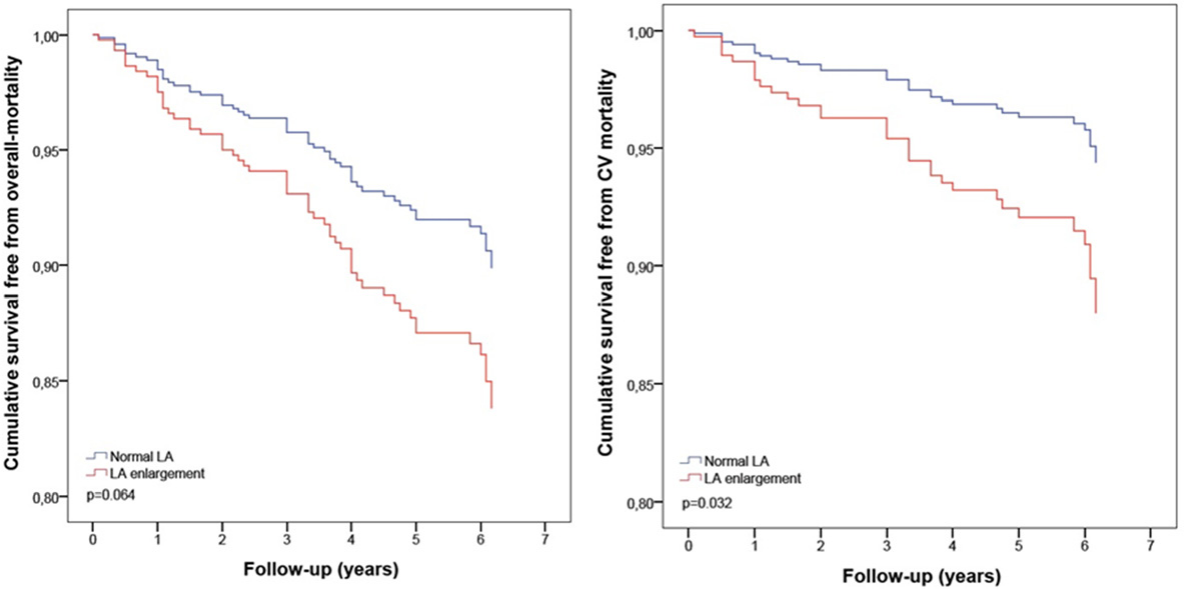
*Left*: Overall mortality and LA enlargement. *Right*: CV mortality and LA enlargement. *LA, left atrium; CV, cardiovascular*

Cardiovascular mortality rate was 2.2-fold higher in patients with LAVi > 34 ml/m^2^. Event-free survival from cardiovascular mortality was significantly lower in these patients (Fig. 4), whilst only a trend towards a higher all-cause mortality was found in patients with LAVi > 34 ml/m^2^ (Table 2).

## Discussion

The present study demonstrates, in a population of 563 patients followed for about 5 years after cardiac surgery, that post-operative LAVi is a long-term predictor of MACCEs and CV mortality, also confirming its prognostic value in predicting MACCEs even after adjustment for age, gender, diabetes, atrial fibrillation at discharge, E/A diastolic index and LVEF.

Left atrium reflects left ventricular filling pressure, a parameter conditioned by structural and functional heart impairement [1, 2]. Consequentely, LA enlargement is an echocardiographic marker that mirrors left ventricular systolic and/or diastolic chronic dysfunction, representing a possible pathophysiological explanation of our results.

Previous studies in patients undergoing cardiac surgery only examined the relationship between LA volume and prevalence of either post-operative atrial fibrillation [13] or symptomatic improvement of HF [14]. Therefore, our results offer novel data highlighting a significant prognostic role of LAVi on MACCEs and CV mortality in such patients. In addition, our study represents a convincing validation of the prognostic significance of the new severity partition cutoffs, suggested by both EACVI and ASE [12], in patients undergoing cardiac surgery. In particular, we showed that moderate and severe LA enlargement is associated with increased rate of MACCEs, while mildly increased LAVi represents a grey zone which does not appear to robustly impact the prognosis.

LA size is a recognized prognostic predictor in primary CV prevention. This was demonstrated in a 8-year follow-up of 5209 individuals included in the Framingham Heart Study, where an increased LA diameter was associated with higher risk of stroke and mortality [15] and other primary prevention studies suggested the independent role of LAVi in predicting CV death [3, 5, 7], MACCEs [5, 7], ischemic stroke [6] and atrial fibrillation [4].

The prognostic significance of LAVi has been confirmed also in secondary CV prevention, especially in patients after acute myocardial infarction (MI). An increased LA volume, assessed within the first 48 h of admission, independently predicted 5-year mortality in a population of 395 MI patients [8]. Similarly, LAVi >32 ml/m^2^ did result an independent predictor of mortality during a mean follow-up of 15 months in 314 MI patients [16], being these results thereafter confirmed in a population of 610 subjects with high-risk MI followed for a mean of 20 months [9]. The relationship between LAVi and CV outcomes was also addressed in 935 outpatients with established coronary artery disease followed for about 4 years, concluding that LAVi was similar to LVEF in predicting mortality and HF hospitalization [17].

The prognostic implications of LA size were confirmed also in patients with valve disease, who did or did not undergo cardiac surgery. Neverthless, the majority of these studies has been mainly focused on post-operative atrial fibrillation [13, 18, 19], LVEF recovery [20] and HF symptoms [14], while harder end-points, such as cardiovascular mortality, had only been evaluated in 176 patients with symptomatic chronic mitral regurgitation undergoing valve replacement by using pre-operative LA diameter [21].

Given that in previous studies the predictive significance of LA size was invariably investigated measuring LA diameter or LAVi with either arbitrary or single cutoff value, our study could represent the first prognostic validation of the new three cutoffs of severity partition, recently recommended by both EACVI and ASE. Our results highlight the importance to quantify the LA size in terms of LAVi; they also suggest that LAVi should be measured not only in routine clinical practice but also in a rehabilitation setting for more accurate risk-stratification and clinical decision making in patients who underwent cardiac surgery.

### Study limitations

The entry criterion for the study was the availability of LAVi measurement and in our registry of 1703 patients, only 563 had such data available for analysis; this approach, driven by the absence of a protocol for LA measurement using LAVi, may have introduced a selection bias.

Although this intrinsic bias could not reflect the real prevalence of LA dimension in the whole population, the rate of LA enlargement, assessed in the entire cohort with LA diameter (cut-off 40 mm), was similar to that evaluated in the subgroup of patients with available biplane left LA volume data (46 % of LA enlargement using LAVi and 48 % using LA dimeter).

Given the retrospective nature of the study, LAVi measurements were obtained by multiple observers working in the same clinical environment and using the same echo machine. This may have created an inter-observer bias related to the variability of the measurements; on the other hand our results would suggest that the prognostic significance of LAVi can be widely applied in the clinical setting.

## Conclusions

Increased post-operative LAVi represents a predictor of adverse outcome after cardiac surgery and provides prognostic information, importantly incremental to several conventional risk factors. The recently recommended LAVi severity cutoffs appear adequate to effectively stratify outcome in patients undergoing rehabilitation after cardiac surgery. These results should be verified trough a prospective, large scale multicentre study design.

## Abbreviations

ACS: Acute coronary syndrome
CABG: Coronary artery by-pass graft
CRP: Cardiac rehabilitation program
E/A: Ratio of the early to late ventricular filling velocities
EF: Ejection fraction
HF: Heart failure
LA: Left atrium
LAVi: Left atrial volume index
LV: Left ventricle
MACCEs: Major adverse cardiovascular and cerebrovascular events
MI: Myocardial infarction
